# Active duty service members' experiences navigating musculoskeletal health throughout their military careers: a qualitative interview study

**DOI:** 10.3389/fspor.2026.1910200

**Published:** 2026-10-28

**Authors:** Brittany F. Hollis, Alexandria B. Gregory, Emily A. Ricker

**Affiliations:** 1 Center for Advanced Research for Military Optimization, Readiness, and Rehabilitation, Department of Neurology and Rehabilitation Medicine, F. Edward Hébert School of Medicine, Uniformed Services University Bethesda, MD, United States; 2 Henry M. Jackson Foundation for the Advancement of Military Medicine, Inc., Bethesda, MD, United States; 3 Department of Military and Emergency Medicine, F. Edward Hébert School of Medicine, Uniformed Services University, Bethesda, MD, United States

**Keywords:** care-seeking, military health system, military personnel, musculoskeletal health, qualitative research

## Abstract

**Introduction:**

Musculoskeletal injury (MSK-I) and pain (MSK-P) pose a substantial burden to Active Duty Service Members (ADSM) and are a leading cause of medical encounters and lost duty days. Evidence indicates that barriers exist to musculoskeletal (MSK) health care-seeking, which can result in exacerbation of MSK conditions (e.g., MSK-I, MSK-P), chronic pain, and greater healthcare costs. The objective of this qualitative study was to interview ADSM about their experiences navigating MSK health during their military careers to inform the development of tools and resources that better equip ADSM and military medical providers to prevent and manage MSK conditions.

**Methods:**

Semi-structured individual interviews were conducted with male and female ADSM (*n* = 60). Interviews assessed ADSMs' experiences with MSK-I/P during military service, the use of military resources to maintain MSK health, and barriers and facilitators to seeking care for MSK-I/P. Interviews lasted 20–60 min, were conducted in-person or over video conference, and were audio recorded and transcribed. Transcript data were analyzed using applied thematic analysis (ATA).

**Results:**

Participants included male (*n* = 31) and female (*n* = 29) ADSM (age=38 ± 10 years, 16 ± 9 years of service) representing all military branches except for the Space Force. The ATA resulted in seven themes: military impact, care-seeking, Military Health System (MHS), prevention and optimization, leadership, mental health, and gender.

**Discussion:**

ADSM revealed that while the military culture promotes physical fitness, it also encourages a “push through pain” mentality, leading to delayed care due to fears of career repercussions or being seen as weak or a burden to one's unit. Additionally, ADSM pointed to certain challenges within the MHS, including long wait times, inconsistent access to specialized care, and a perception that some providers dismiss ADSM pain. Results revealed additional areas of investigation and intervention, such as a bidirectional link between MSK-P and mental health, and identified gender-specific challenges, such as a lack of resources for women's health issues like postpartum MSK conditions. Overall, the findings identified opportunities for systemic and cultural changes to destigmatize MSK issues, prioritize prevention and proactive care-seeking, and improve healthcare access for MSK conditions during military service.

## 1 Introduction

Musculoskeletal injury (MSK-I) and pain (MSK-P) represent the leading cause of military medical encounters and limited duty days among US Active Duty Service Members (ADSM) and lead to high rates of healthcare seeking among Veterans (1, 2). This burden increases with age and is disproportionately higher among female ADSM and Veterans (2–6). However, the true burden of MSK-I/P among ADSM is likely underestimated due to underreporting. An estimated 64% of military recruit MSK-I and half of MSK-I in operational units go unreported to medical personnel (7–9). Key drivers of non-reporting include fear of duty-limiting profiles [i.e., documentation of duty limitations due to medical condition(s)], career repercussions, and logistical inconvenience (8, 9). Delayed treatment may accelerate the progression from acute MSK-I to chronic MSK conditions, compounding the long-term health impacts and healthcare expenditures across both the Military Health System (MHS) and Veteran's Health Administration (VA), not to mention the health burden for the individual. As such, understanding unique barriers ADSM face in accessing care could highlight areas where resources, programs, or policies about medical care-seeking for MSK conditions could be improved.

While quantitative research approaches (e.g., chart reviews, surveys) have characterized MSK epidemiology and population-level risk factors (10, 11), they offer limited insight into systemic access barriers, organizational constraints, and the lived experiences of ADSM. Qualitative research is uniquely suited to capture these complex cultural and structural dynamics, yet qualitative evidence regarding MSK health experiences among ADSM remains sparse, particularly for female ADSM. Previous qualitative research from Army (2008, 25% female) and Marine Corps (2017–2019, male only) cohorts reveal consistent challenges, including administrative and logistical hurdles, variable care quality, pressure to “suck it up,” and the motivation to finish training courses at all costs (12, 13). The thematic alignment from these two cohorts, distinct with respect to service branch and time examined, indicates that more work is needed to overcome challenges accessing MSK care during military training and service.

To address this need, the aim of this study was to conduct qualitative semi-structured interviews with male and female ADSM to gain a more comprehensive understanding of their experiences with MSK health throughout their military careers. Researchers from the VA previously conducted interviews regarding MSK health experiences among women Veterans (14); the current study is an analogous effort in ADSM, including males and females. Ultimately, the goal is to combine insights garnered from qualitative investigations in both cohorts to inform the development of tools and resources for ADSM and Veterans to improve MSK health and healthcare.

## 2 Materials and methods

### 2.1 Study design

This qualitative study used semi-structured individual interviews to examine ADSMs' MSK health experiences and subsequent care seeking behaviors during their military service. The study was grounded in a pragmatic paradigm, treating participant accounts as context-dependent descriptions of experiences with MSK health and care-seeking. Rather than seeking an absolute truth, this stance prioritizes generating actionable knowledge to inform practical improvements within military health systems.

### 2.2 Study setting, sample, and recruitment procedures

Participants were recruited from Naval Support Activity Bethesda (Bethesda, MD), Walter Reed National Military Medical Center (Bethesda, MD), Uniformed Services University (Bethesda, MD), and Alexander T. Augusta Military Medical Center (Fort Belvoir, VA) through fliers and email announcements that were intended to target a wide range of ranks, unit types, and demographic groups. Participants were screened for eligibility via online form and telephone screen. To be eligible, participants had to be Active Duty with a history of MSK-I/P during military service. Requiring prior MSK-I/P was an intentional eligibility boundary designed to ensure that participants had direct experience relevant to the interview questions and research objective. An information sheet was sent to potential study participants prior to the interview so that they could review the study purpose, procedures, risks and benefits, including that participation was voluntary and researchers would take precautions to maintain participant privacy and confidentiality. Before the interview, a brief reiteration of the study purpose was provided and the interviewer confirmed verbal consent before proceeding. Interviews were conducted in-person at the Uniformed Services University in a private office (*n* = 12) or virtually (*n* = 48) using Google Meet (Google, Inc) by a civilian researcher in plain clothing, away from any military leaders. All interviews were conducted between November 2024 and June 2025. Interviews took place based on participant's convenience and participants were not asked whether they were taking part during duty or non-duty hours. The interviews lasted approximately 20–60 min. The study was reviewed by the Human Research Protections Program at the Uniformed Services University and received an exempt determination under the provision of 32 CFR 219.104(d)(ii).

Participants were recruited using a purposive quota sampling strategy combined with volunteer self-selection to ensure representation of both male and female ADSM with lived experience of MSK-I/P during military service. The target sample size was established *a priori* as 60 participants, with a goal of approximately 30 male and 30 female participants. This target was selected to provide information-rich interviews overall while also allowing adequate depth within each sex subgroup, given the investigators' interest in understanding potential gender-specific experiences with MSK health and care-seeking. The planned sample size was informed by empirical methodological research on saturation in qualitative interviews. Prior research suggests that code saturation, or identification of the range of thematic issues within a dataset, may occur within approximately 9 interviews, whereas meaning saturation, which reflects a more nuanced understanding of the dimensions and variation within themes, may require approximately 16–24 interviews (15). Other empirical work has similarly demonstrated that approximately 16 individual interviews may capture 90% of thematic content (16). Therefore, targeting approximately 30 participants within each sex subgroup was expected to exceed commonly reported thresholds for thematic saturation while providing sufficient depth to explore variation in experiences within and across male and female participants. The approximately equal representation of male and female participants was also an intentional design feature intended to preserve the ability to conduct sex-stratified analyses and facilitate potential comparisons with related research being conducted with women Veterans (14). Saturation was not formally assessed as a stopping criterion during data collection; rather, data collection concluded when the prespecified recruitment target was reached. The final sample included 60 participants (31 male and 29 female), closely approximating the planned sampling distribution.

### 2.3 Qualitative interviews

The interview guide for this study was developed collaboratively by three members of the research team (EAR, BFH, ABG) with input from clinicians and researchers with similar subject matter expertise (Supplementary Material). Questions were developed to better understand ADSMs' experiences with MSK-I/P during military service and to understand their MSK care seeking behaviors. Specifically, participants were asked about their MSK health and well-being, perceptions of DoD services and programs, and facilitators and barriers to MSK health and care. The interview guide remained unchanged throughout the course of the study. Some interview questions were tailored based on the participant's reported service branch, which was ascertained during pre-screening [e.g., Do you use any programs or services offered by (insert branch here) or the military/DoD to help you to maintain or improve your MSK health?] A health services researcher (BFH) conducted all of the pre-screening and interviews, and thus was aware of the participant's age, sex, and service branch prior to the interview. All interviews were audio recorded and transcribed using AI transcription (Rev.com, Inc). To ensure transcript accuracy, the interviewer (BFH) reviewed each transcript in its entirety while listening to the corresponding audio recording. Discrepancies between the AI-generated transcripts and audio recordings were manually corrected prior to qualitative analysis. Participants were assigned a randomly generated study identification number at the time of screening, and all direct identifiers were removed from transcripts prior to analysis. Audio recordings and transcripts were maintained on DoD-approved computer storage protected by two-factor authentication, with access restricted to members of the research team. Following verification and deidentification, transcripts were imported into NVivo 14 (Lumivero, 2024) qualitative analysis software.

### 2.4 Data analysis

Transcripts were analyzed qualitatively using applied thematic analysis (ATA), an inductive approach to analyzing qualitative data that identifies, describes, and interprets patterns of meaning (17, 18). ATA was appropriate for this applied health-services question because the goal was to generate a systematic, actionable account of barriers and facilitators to MSK health and care-seeking rather than to develop a formal theory. ATA was selected because its framework allows for both inductive and deductive coding. This approach is well suited for identifying salient patterns across participant accounts while maintaining direct relevance to real-work healthcare practice and implementation. We first conducted open coding. Three researchers (EAR, BFH, ABG) identified key concepts emerging from the language used by participants and assigned codes to segments of text. All three researchers independently coded the first five transcripts and then compared their coding and interpretations to develop the preliminary codebook. To assess consistency in the application of the preliminary codebook, coding agreement was examined at the level of coded text segments across these five transcripts. An aggregate Cohen's *κ* = 0.86 was calculated across all three researchers. This *κ* value was used as a descriptive indicator of coding consistency during codebook development rather than as a criterion for establishing the validity of the thematic analysis. Following the coding comparison, differences in code application and interpretation were discussed by the research team and resolved through consensus. The codebook was subsequently refined before coding of the remaining transcripts proceeded.

Several procedures were used to promote reflexivity and reduce the influence of any single researcher's assumptions on the analysis. Throughout analysis, the researchers met regularly to discuss coding decisions, emerging concepts, potential new codes, and alternative interpretations. The codebook was iteratively refined, with all revisions documented, and the research team maintained a documented trail of qualitative analytic decisions throughout the analysis. These procedures provided an audit trail of the analytic process and opportunities for peer debriefing and reflexive consideration of how researchers' disciplinary backgrounds, prior experiences, and assumptions could influence interpretation of the data. Following completion of coding, the researchers worked together to identify themes, subthemes, and sub-subthemes.

To ensure methodological rigor, we implemented targeted trustworthiness strategies. Credibility and dependability were supported through analyst triangulation (three researchers independently coding initial data and engaging in regular analytic meetings), an iterative coding and codebook refinement process, and grounding of all final themes in illustrative participant quotations. Confirmability was enhanced by collaborative review of coding and theme decisions rather than reliance on a single analyst. Finally, transferability is supported through detailed description of the sample, military context, and study setting, enabling readers to evaluate the relevance of findings to other settings and populations.

The research team brought complementary backgrounds in qualitative methodology, military health research, and MSK health to the study. BFH, a PhD-trained health psychologist and health services researcher, served as the primary interviewer and qualitative methodologist. BFH has extensive experience conducting and analyzing qualitative research, including ATA, as well as substantial experience conducting research with military and Veteran populations, including research positions within the Veterans Health Administration and Defense Health Agency. BFH is a civilian researcher with no personal history of military service. ABG has a MA in kinesiology with years of experience conducting military health research, specifically examining MSK care-seeking behaviors in ADSM. ABG is a civilian researcher and data manager with no personal history of military service. EAR has a PhD in kinesiology and several years of experience conducting military health research, especially pertaining to MSK health in ADSM. Additionally, EAR has conducted previous qualitative research in ADSM related to MSK health, care-seeking, and gender stigma, all themes that arose in the current study. EAR is a civilian researcher with no personal history of military service. None of the researchers had an established relationship with participants prior to their enrollment in the study, nor did the researchers hold clinical positions in the MHS. The researchers recognized that their professional backgrounds, prior research experiences, and relationships to the military context could influence the questions they prioritized and their interpretation of participants' accounts. In particular, BFH's extensive professional experience conducting research within military and Veteran health settings provided familiarity with military health systems, terminology, and culture, while her position as a civilian researcher meant that she did not share participants' lived experience of military service. This combination of familiarity with the research context and an outsider perspective was considered throughout data collection and interpretation.

Basic demographic information gathered during the pre-screening and interview (age, service branch, military occupation, and years of military service) was summarized descriptively. Military occupations were categorized using the framework described by Jannace et al. (2023), which adapts the DoD Occupational Classification System to aggregate service branch-specific specialties into standardized functional categories (19). Military rank was not collected. As such, occupational categories could not be distinguished between enlisted and officer roles and were consolidated into five occupational categories: Infantry and Tactical Operations, Communications and Intelligence, Health Care, Functional Support and Administration, and Engineering and Maintenance) to facilitate baseline sample reporting (Table 1).

**Table 1:** Participant descriptive characteristics.

Characteristic	All *N* = 60 n (%)	Male *n* = 31 n (%)	Female *n* = 29 n (%)
Age (yrs)^a^			
≤25	7 (11.9)	3 (10.0)	4 (13.8)
26–30	8 (13.6)	3 (10.0)	5 (17.2)
31–35	8 (13.6)	4 (13.3)	4 (13.8)
36–40	13 (22.0)	4 (13.3)	9 (31.0)
≥41	23 (39.0)	16 (53.3)	7 (24.1)
Years of Service^b^			
0–4	5 (8.8)	1 (3.4)	4 (14.3)
5–10	13 (22.8)	6 (20.7)	7 (25.0)
11–20	24 (42.1)	9 (31.0)	15 (53.6)
≥21	15 (26.3)	13 (44.8)	2 (22.8)
Service Branch (n (%))			
Army	22 (36.6)	11 (35.5)	11 (37.9)
Air Force	14 (23.3)	7 (22.6)	7 (24.1)
Marine Corps	5 (8.3)	3 (9.7)	2 (6.9)
Navy	15 (25.0)	8 (25.8)	7 (24.1)
Public Health Service	2 (3.4)	1 (3.2)	1 (3.4)
Coast Guard	2 (3.4)	1 (3.2)	1 (3.4)
Military Occupational Category^c^			
Infantry and Tactical Operations	4 (6.9)	3 (10.0)	1 (3.6)
Communications & Intelligence	6 (10.3)	4 (13.3)	2 (7.1)
Health Care	37 (63.8)	16 (53.3)	21 (75.0)
Functional Support & Administration	9 (15.5)	5 (16.7)	4 (14.3)
Engineering and Maintenance	2 (3.4)	2 (6.7)	0 (0)

a Male *n* = 30; ^b^Male *n* = 29, Female *n* = 28; ^c^Male *n* = 30, Female *n* = 28.

## 3 Results

### 3.1 Participant characteristics

The median age of the participants was 38 years (range 22–57 years) and participants had served a median of 16 years (range 2–38 years). They represented all branches of the US military except the Space Force and reported a variety of military occupational categories, though Health Care was the most frequent category (64%) (Table 1). The relatively long average time in service should be considered when interpreting the findings because participants with longer careers may have accumulated more experiences with MSK conditions, military culture, leadership, and the MHS than more junior service members. Additionally, the preponderance of health care occupations should be taken into account when interpreting findings, as these participants may be more health literate and have different experiences navigating health care compared to those not working in a health field. Importantly, approximately 20% of the participants reporting a health care occupation were medical or clinical psychology students, reflecting different exposures to the MHS compared to physicians and medics.

### 3.2 Themes

The ATA resulted in seven main themes: military impact, care-seeking, Military Health System, prevention and optimization, leadership, mental health, and gender. Each theme is defined below with descriptions and elaborations organized by subtheme. Block quotes in the text are often abbreviated, though care was taken to ensure meaning was retained. Sub-subthemes and illustrative interview quotes are shown in Table 2**.** Theme, subtheme, and sub-subtheme definitions are in Supplementary Table 1.

**Table 2:** Themes, subthemes, and illustrative quotes from interviews.

Themes	Illustrative Quotes
Care-Seeking	A combination of personal, cultural, and systemic factors that influence one's decision to seek care or not seek care for MSK issues
Subtheme: Barriers	
Sub-Subthemes:	
Inconvenience	“…the hours available for physical therapy or any of the resources…it's always the travel time. And then lack of consistency and schedule…if I could book I guess specific time each week and keep that, or even every other week, that would be great. And I could work it into my work schedule. But having to move it around all the time and then try and work around my meetings is very challenging.” [Participant 37]
Career	“And there's a lot of people that tend to keep a lot of the issues quiet, especially in very high tempo units because if they don't, there is an implicit fear that they will lose their flying status or their ability to go out and do missions and there's some hesitancy to go seek provider care.” [Participant 14]
Personal	“It took a lot for me to learn how to self-advocate. And if you don't in the military, it's so easy for your [primary care manager] to dismiss you. It took a lot for me, especially even with that plantar fasciitis. It was like, it's just your foot. Suck it up. Let's go. Come on. What's wrong with you?” [Participant 26]
Culture	“…there's still that stigma of, oh no, I'm fine. I can fight through it. I'm not going to go see medical for this. And if you go to medical too much, you could be labeled as a malingerer as you're not pulling your weight, oh, you're going to medical again. And then, so I think that because of that stigma, you have the people fighting through injuries.” [Participant 26]
Provider	“And basically they were so busy that they just turned me away. Then I was like, well, this is annoying. And I just kept going to classes and over time it got better. But then ever since that, it was really loose, so it just started to dislocate it all the time. And then I think I was just kind of upset from that first experience… I just dealt with it on my own. I was like, they’re just going to turn me away.” [Participant 34]
Subtheme: Facilitators	
Sub-Subthemes:	
Access	“The provider had an office in our squadron building and he was there at least once a week, sometimes more. And it was easy for me to be able to walk into his office and be like, ‘Hey, my back's hurting. What can we do? How can we talk about it? What things can we continue to do?’ And then I could continue to follow up with him there rather than having to take the time, go to the clinic, schedule an appointment and work through all of the bureaucracy, for lack of a better term with the medical system.” [Participant 14]
Culture	“Yeah, so because we are more health inclined or aware, we encourage service members to take care of themselves, take care of your mental health, take care of your physical health.” [Participant 2]
Leadership	“My chain of command has helped me a lot and advocated for me to get…seen by a specialist” [Participant 26]
Personal	“I'm not a hypochondriac, but if I have an issue, I'm going to go see a doctor…I don't tough it out. If you want to tough it out, go for it, but I'm not going to tough it out…There are doctors and I get free healthcare, so I'm going to take advantage of it.” [Participant 3]
Subtheme:	
Personal Decision about Care-Seeking	“I met people that had multiple injuries, and so having seen what they went through, and even just people who didn't go and be seen when they were injured in jobs, I was like, ‘yeah, that's not me.’ I was like, ‘if I get injured, I'm immediately going over there. I'm telling ‘em, ‘Hey, yeah, I got hurt. Let's get this documented.’ Because at the end of the day…if there is ever a contention of like, ‘Hey, was this on the job or wasn't it?’ I'm like, ‘it's right there, brother. It's right there.’ I came in literally the day after.” [Participant 4]
Military Impact	The effect of military service on MSK health
Subthemes:	
Pain	“The pain is pretty constant…my leg is always tingling…some days are worse than others…there's definitely been times where I just moved the wrong way and I don't know what happened, but I made something very angry at me. And then I'm down for the count.” [Participant 1]
Service-related issues	“I think it's a combination of the gear that we're issued or given, whether it's the boots aren't the right support for the individual, the packs that we wear are not shown to us how to properly wear them for our height or our weight. All of those factors come into play. How we load out our packs, how we load out our gear. And then for the healthcare workers wearing those lead aprons, are we wearing the right shoes?” [Participant 23]
Culture	“It was a competition between people to see how little sleep they could get, and on top of that, how much they were able to still work out or how many times they hit the gym instead of sleeping. And I think there's definitely competitiveness that I think it might just be inherent in people who are drawn to the military that type A personality that wants to be the best.” [Participant 26]
Prioritization	“And so finding that kind of balance between, at the end of the day, the mission's going to come first, no matter, regardless of what the injury is…So I think the biggest thing is to depend on what kind of command you're at. And it depends what the mission is of that command.” [Participant 1]
Career	“there's a lot of people that tend to keep a lot of the issues quiet, especially in very high tempo units because they don't, there is an implicit fear that they will lose their flying status or their ability to go out and do missions and there's some hesitancy to go seek provider care.” [Participant 14]
Military Health System	The combination of access, quality, and patient experience within the healthcare system
Subthemes:	
Quality	“The person that I saw was just a family care practitioner. And I mean, I think the diagnosis of a shoulder impingement is not that challenging. I mean, I think I could diagnose myself with shoulder impingement, but she's not an orthopedist or anything like that, or a chiropractor that could figure out what was going on…and there's no [physical therapy], no follow up.” [Participant 3]
Patient opinion of provider	“I felt like I was definitely dismissed, felt almost like I was wasting her time…and the fact that I was being questioned as far as I didn't know what my pain was with the symptoms I was having if they were real. And kind of having that kind implied to me as if I basically saying, ‘I don't think you're telling me the truth’… normally I pushed through stuff so for me to be at the point where I'm like, ‘I need some help.’ That's kind of me opening up like, ‘Hey, I'm vulnerable. I have an issue, help me.’ So I really didn't appreciate it.” [Participant 1]
Access	“And so if there was an easier way to just be message, ‘Hey, I'm having a weird pain, I would like to go see a sports med doc,’ I would probably be more open to doing it that way rather than having to go in and take an appointment slot from a primary care physician” [Participant 31]
MHS to VA transition	“Because right now it's all about the almighty dollar. And it seems like the feeling is it's cheaper to just push the problem to the VA and let the VA deal with it after the service member exits than it is to spend the money to get a diagnosis and fix it on the front end.” [Participant 52]
Prevention and Optimization	The systemic and individual barriers and facilitators to avoid MSK injury/pain and maintain MSK health
Subthemes:	
Resources	“I think that what they have already needs to be expanded. So we have the H2F teams, but as of right now it's like a team of four people for 7,000 people. And that's just not near what it needs to be to really make a big difference. I also think that people who are the trainers at the beginning of the military careers need more training about all of that. Just the advice that you hear drill sergeants, giving these new soldiers is like, no, don't do that. That's terrible. That's a really great way to screw up your knees forever. Don't do that. And so I think that there needs to be a knowledge base with the people that have first contact.” [Participant 18]
Culture	“When I went out one of my deployments, I'm being more exposed to a lot of young folks and what blew my mind was I'm sitting at one of these gyms and there's young men, young women with these flip pads and they're writing down their exercise or their routines. They are actually managing their physical fitness…it gives ‘em goals yet at the same time helps make sure they're avoiding injuries…And I see the culture is trying to say, suck it up and drive on. It may not be the best thing. So I do see those as positive things compared to what I saw 40 years ago.” [Participant 7]
Prevention vs. reaction	“But I think that a lot of unit training, physical training stuff for PT, I think a lot of that could be restructured and reorganized to be more beneficial for the body, while also mitigating risk for sustaining a musculoskeletal injury. So I think some people are taking a more proactive approach, but I think the general, generally speaking, I would say it's reactionary and it's not prioritized until it's a problem.” [Participant 1]
Intrinsic motivation	“…knowing what I have to do for myself. I guess knowing my body and knowing that, ‘Hey, if I need to get up and run six miles today, I need to go do that because it's going to keep me healthy. It's going to hurt a little bit.’ But so it's finding those windows of time to stay healthy and also not worry about, I guess being the strongest guy in the room because it's not always about that. I kind of focus on maintaining my weight to a proper weight so I'm not overbearing my body unnecessarily. And then just keeping my cardio and that stuff at the highest I can keep it for endurance purposes.” [Participant 20]
Leadership	The influence of military leadership on MSK health prioritization and care
	“This guy had a letter from his physical therapist. He was seen out in town and it was a very detailed letter talking about his diagnosis, his signs and symptoms, his treatment plan, and then recommendation for 30 days light duty. That's what the physical therapist said. And I watched his medical officer read that, read that letter, and he said, '30 days light duty, huh? Well best I can do is five.' I was just kind of just shocked…if you don't enable someone to heal, when are you going to, I mean, they're not going to get better” [Participant 1]
Mental Health	The bidirectional relationship between mental and MSK health
	“Not just for my physical health or for my mental health, right? Because I was an active person and then when I couldn't do the thing, I didn't have anything else to fill to put in that void. And so I was definitely not enjoying life as much.” [Participant 28]
Gender	Gender-specific experiences and bias in MSK health and care
	“And I've talked to a lot of soldiers and just women in the military period. And it's a very common theme. And I tell all my young soldiers, you've got to advocate for yourself. If you know something is wrong, switch providers, demand to go out in the civilian clinic, it is horrible that you have to chase it. But if you know that you need something else you are not getting, keep advocating for yourself and don't take “no” for an answer. Find a doctor that's going to listen to you, keep switching, and, excuse me, it's so hard for our young soldiers, our young female soldiers that don't want to be a problem. They don't know how to talk to, especially the military doctors that are majors and lieutenant colonels and colonels, especially when they're not being listened to. But I find that it is a reoccurring, but I think some of it is doctors, some of it is just western medicine in general.” [Participant 54]

#### 3.2.1 Theme 1-military impact: the effect of military service on MSK health

##### 3.2.1.1 Subtheme: pain

Several participants discussed chronic or persistent MSK-P associated with their military service that severely affects daily physical functioning (e.g., inability to walk). Additionally, participants mentioned constant MSK-P due to previous MSK-I, or MSK-P that was distracting, debilitating, or cyclical.

##### 3.2.1.2 Subtheme: service-related issues

Participants discussed direct and indirect physical and operational demands of military service that they perceived as impacting their MSK health. These demands varied based on required military duties and military operational specialty (MOS). Participants specifically mentioned heavy load carriage demands and equipment such as boots and body armor taking a toll on their MSK system. Others referenced military living conditions as hindering their MSK health, such as cramped spaces and hard surfaces for those working on ships, rigid airplane or helicopter seats for those operating aircraft, and uneven, unpredictable terrain for those operating or training on land.

[Ship] racks [are] not the most comfortable thing…the staircases on the ship [are] steel…I constantly have to bend to get through spaces…it was rough on the back muscles. [Participant 2]

Participants expressed that high operational tempos of their military occupations limited time for recovery or made it difficult to get time off from work for medical appointments or physical recovery. In several accounts, these perceived barriers contributed to participants having a history of untreated MSK-I or deploying with known MSK-I. Additionally, the term “wear and tear” was used by some participants, reflecting a belief that military duties may inevitably result in suboptimal MSK health. Furthermore, rigorous physical training was perceived to negatively affect MSK health, especially if ADSM were struggling to meet physical fitness or weight requirements and subsequently developed maladaptive or excessive physical activity habits without proper recovery built in.

Conversely, some participants reported positive effects of military service and physical training on MSK health. For these participants, the requirement to pass regular physical fitness tests instilled physical activity habits that contributed to sustained health.

They've really updated and adapted stretching and recovery techniques…I appreciate the…warmup and the recovery system so much that I've incorporated [it] into my personal physical fitness. [Participant 33]

##### 3.2.1.3 Subtheme: culture

Interviews consistently highlighted participants' perceptions of a military culture that promotes physical competitiveness and a “push through pain” mentality. Participants mentioned how the team mentality of the military can aid in keeping individuals active, as group training can boost motivation and adherence. On the other hand, some participants described how the highly competitive environment led individuals to try to lift heavier weights than is appropriate for their fitness level and/or use poor lifting form, which participants felt contributed to preventable MSK-I. Furthermore, participants noted a pervasive perception that an ADSM is undervalued when they are injured or in pain or are unable to operate at the same high level.

There's a lot of ego lifting…people that just…try to push [their] body…And that was sort of praised rather than used as an example of what not to do. [Participant 27]

##### 3.2.1.4 Subtheme: prioritization

Participants described experiencing a tension between mission readiness and individual well-being were often conflicting priorities, which they felt shaped their MSK outcomes. For example, some participants reported choosing to deploy with known MSK-I. Additionally, participants referenced having daily job requirements that they felt interfered with the time needed for physical training to maintain MSK health. Participants were divided in whether they perceived that the DoD placed a high enough value on MSK health, suggesting that MSK health prioritization may depend on your service branch and/or MOS.

All this money they dump into creating…a new [physical training] program …where's it in practice?…We spend all this money, but then we don't do anything to change how we treat the Soldiers. [Participant 20]

Many participants also expressed the perception that the DoD does not sufficiently prioritize specialty care in the MHS (e.g, sports medicine, physical therapy, occupational therapy) or holistic care (e.g., nutrition, acupuncture, chiropractor, massage), services they believed could improve MSK health.

We don't really have family medicine practitioners or sports medicine practitioners…[clinics] don't have a sports med physician for these athletic trainers to work under their license. [Participant 1]

Individual decisions regarding the timing of care seeking were also influenced by training or operational milestones, as participants often prioritized completing a certain assignment or training course prior to obtaining medical care. Specifically, some participants discussed perceiving that their providers offered temporary measures, or “quick fixes,” like pain medication or injections, primarily to get them through a training course/phase or deployment.

##### 3.2.1.5 Subtheme: career

Participants reported intentionally avoiding drawing attention to MSK-I/P due to concerns over losing their eligibility to serve or perform certain duties, such as flying, either temporarily or permanently. Additionally, participants recalled observing colleagues undergo the Medical Evaluation Board (MEB) process, the formal administrative system used to evaluate whether a ADSM meets the medical retention standards, which participants perceived as a significant threat to career longevity.

[There are] a lot of people that tend to keep a lot of the issues quiet…there is an implicit fear that they will lose their flying status or their ability to go out and do missions and there's some hesitancy to go seek provider care. [Participant 14]

#### 3.2.2 Theme 2-care-seeking: a combination of personal, cultural, and systemic factors that influence one's decision to seek or not seek care for a MSK condition

##### 3.2.2.1 Subtheme: barriers

Participants described various perceived barriers to MSK care, including logistical hurdles, fear of professional repercussions, mistrust in the medical system, and social stigma. In terms of logistical challenges, participants highlighted the inconvenience of care, such as lack of geographical proximity to needed medical resources or specialists needed, perceived difficulties taking time off work to seek care, and long wait times between initial appointment requests and provider availability.

You got to go see your [Primary Care Manager] first…now you got to make another [specialist] appointment…it's probably like four appointments in before you really get after some real care to help it. [Participant 28]

Some participants expressed concern regarding the negative impact of MSK care-seeking on one's military career, which frequently led them to to prioritize professional advancement and a sense of duty over their own physical well-being. Other personal, individual-level barriers were described as preventing care-seeking, such as the perceived burden of having to self-advocate to receive care or the belief that care would be ineffective.

I felt like a lot of the doctors that I was getting care from really didn't care. They were just kind of there doing a job…to get me back to physically fighting shape. [Participant 44]

Finally, participants described societal and military cultural norms as barriers to care, such as social stigma associated with being injured or seeking care, a fear of letting one's team or unit down if injured, and a perception that lower ranking ADSM face greater obstacles when accessing care than higher ranking ADSM due to the hierarchical leadership structure within the military.

Weakness is a sin. Not being part of the team…is a sin, you don't want to be that guy…I got to take care of myself falling apart and the guilted craziness against that kind of culture. [Participant 45]

##### 3.2.2.2 Subtheme: facilitators

Facilitators for care-seeking described by participants included a mixture of medical service availability, and geographical, social, and systemic factors. Participants suggested medical service availability, or having more accessible care, whether from a geographical or logistical (e.g., time and resource availability) standpoint, promoted their willingness to seek care. For instance, some participants reported that certain units provided ready access to an athletic trainer to help with acute injury management.

The [Special Operations Forces] community, they got physical therapists on standby… athletic trainers, all these people are ready to go. So the slightest injury sparks up, they have someone they can get them back on track as expeditiously as possible. [Participant 1]

Participants also described unit-level cultural factors that facilitated care-seeking, highlighting instances where supportive leadership and shared unit norms encouraged medical utilization. Finally, several participants cited past positive interactions with providers as a key factor that increased their personal comfort and likelihood of seeking care for MSK issues in the future.

##### 3.2.2.3 Subtheme: personal decision about care-seeking

Some participants highlighted internal motivators, noting that their decision-making about seeking care included a personal willingness to seek prompt care when needed. Conversely, others recognized their personal decision to put off care despite thinking that it would be helpful, with some expressing regret and wishing they had sought care sooner.

I got injured, I was in training, so I was like…I'm not getting seen…[I'm graduating…I would say that probably for 98% of my injuries, I definitely did not seek care when I should have. [Participant 1]

Personal perceptions of pain also strongly influenced participants' care-seeking decisions. Participants noted factors such as one's own pain threshold or tolerance, a personal decision to push through pain, self-evaluations to delineate between pain that requires care versus pain that will likely resolve on its own, and pain that affects daily activities and military duties, thus requiring care. Additionally, several participants reported choosing to delay or avoid surgery that was recommended by providers or that would eventually be necessary. Finally, a few participants noted that they sought care specifically to ensure proper documentation of their MSK condition for future disability status after leaving active duty. Within this cohort, these varied personal considerations shaped distinct individual care-seeking decisions.

I still have enough range of motion where this is just a strain. I'm just going to ice it… I'll take an NSAID and then I just deal with it for a few weeks until it goes away versus, okay, this is bad enough. [Participant 29]

#### 3.2.3 Theme 3-Military Health System: the combination of access, quality, and patient experience within the healthcare system

##### 3.2.3.1 Subtheme: quality

The interviews highlighted variations in the perceived quality and standards of care across the MHS. Participants shared several personal accounts of consulting non-MSK specialty providers (e.g., primary care, family medicine) as their mandatory first line of care, with some expressing a belief that primary care providers would benefit from additional MSK-specific training, such as enhanced evaluations for common MSK-I. Some participants characterized their care as receiving temporary remedies, or “quick fix” treatments, that didn't address the root cause of their issues, and receiving no follow-up post-diagnosis to ensure healing. Additionally, several participants speculated, particularly when reflecting in hindsight on earlier career stages, that senior rank might be associated with improved healthcare access and quality.

I am a higher rank [so] I think I get a little bit different treatment…I'm able to get to the front of the line a lot. But…even for me…I had to go through a whole bunch of extra hoops to get in [Participant 43]

##### 3.2.3.2 Subtheme: patient opinion of provider

Although some participants referenced positive provider interactions, many accounts reflected feelings of dissatisfaction with care. Participants attributed this dissatisfaction to feeling dismissed by their provider, perceiving that their provider didn't believe their reported pain and symptoms, or feeling that providers suspected them of exaggerating their symptoms or malingering.

Soldiers are coming in with injury and being treated as though they're trying to malinger… that doesn't excuse not taking care of Soldiers because there are people that realistically do have very severe or difficult things they're dealing with. [Participant 4]

Additionally, one African American female participant shared her perception that, due to her race, providers did not take her pain as seriously and interpreted her care-seeking as drug-seeking rather than evaluating her MSK conditions.

##### 3.2.3.3 Subtheme: access

As discussed in the care-seeking theme, participants reported that access to care was often variable, with barriers such as long wait times perceived as hindering timely utilization. However, some participants specifically acknowledged that military service provided enhanced access to medical care through the MHS compared to their perceptions of civilian medical care, though many expressed a desire for more providers or support for providers to meet the demand for care. Along these lines, participants advocated for greater access to specialty care and holistic resources to support MSK health (e.g., sports medicine, chiropractors, dietitians, acupuncture, athletic trainers, strength and conditioning coaches). Participants also expressed frustration with navigating referral systems for specialty care, which they felt unnecessarily prolonged care and treatment timelines.

Furthermore, some participants attributed administrative delays and care limitations to policies associated with TRICARE, the military health insurance provider, voicing concerns that billing constraints negatively impacted their patient experience. In addition, several participants reported challenges finding civilian providers who accept TRICARE, which they felt resulted in logistically inconvenient care or out-of-pocket expenses for out-of-network providers.

You can't get seen for your foot and your knee on the period on the same day because of billing…so much of my time is eaten up going to physical therapy for multiple parts of my body that could be assessed and done at the same time. [Participant 49]

##### 3.2.3.4 Subtheme: MHS to VA transition

Some participants expressed a perception that their providers sometimes sought to “pass the buck” to the VA rather than treating their MSK-I/P entirely while on Active Duty. At the same time, several participants emphasized their belief in the importance of having clinical documentation of their MSK-I/P during service to ensure future eligibility for VA disability compensation upon separation or retirement. However, a few participants alleged that some individuals exploit this process by inflating documented medical conditions to maximize disability ratings, regardless of clinical merit.

Folks at the end of the line will ask you, well, why didn't you bring any of this up while you were having the issue? And that's kind of an unfair question and really demeaning to ask someone who's done exactly what [is needed], to deploy, to be deployable…. Knowing full well that if you don't, certain parts of the mission fail [Participant 62]

#### 3.2.4 Theme 4-prevention and optimization: the systemic and individual barriers and facilitators to avoid MSK injury/pain and maintain MSK health

##### 3.2.4.1 Subtheme: resources

Participants reported that the resources available to prevent MSK-I/P and optimize fitness and support optimal MSK health varied by location, service branch, or unit. Participants acknowledged that having access to quality fitness resources (e.g., fitness centers and pools) and MSK health resources (e.g., athletic trainers, physical therapists) was beneficial, though they noted these assets were not uniformly available across all duty sites. Even when resources were available, participants reported that they were not always utilized, sometimes due to logistical inconvenience (e.g., crowded gyms, limited hours). When asked specifically about DoD-provided resources for MSK health, participants indicated that resources were either unavailable at their site or remained unused due to a perceived lack of efficacy. Many spoke positively about the Army's Holistic Health and Fitness (H2F) program, while noting perceived limitations, such as variability in implementation across locations and a belief that other service branches lacked comparable programs. Additionally, several participants emphasized the need to increase awareness of available resources to encourage broader utilization.

My last unit…had a full out, blown out H2F team that was highly utilized…we had specialized athletic trainers, physical therapists and stuff like that on our team to help rehabilitate those injured soldiers back to a hundred percent. [Participant 39]

Other participants' suggestions for MSK prevention and optimization included providing ergonomic office equipment for those with sedentary assignments and expanding access to exercise recovery modalities (e.g., yoga, stretching, low impact). Finally, participants cited budget constraints as a key factor limiting the availability of these resources.

##### 3.2.4.2 Subtheme: culture

On average, participants had been serving in the military for over 15 years and were therefore able to speak to their perceptions of the evolution of military fitness culture and shifting approaches to MSK health. Participants pointed to initiatives such as training flight instructors on MSK health, stretching and dynamic warm up programs, the holistic efforts associated with H2F, and changes in physical fitness tests intended to better align with functional fitness needs of ADSM. One participant observed that younger generations of ADSM appeared to more actively managing their fitness in the gyms by planning, tracking, and documenting their workouts to meet fitness goals and avoid injury.

The fact that people want to be at the top of their game and with the holistic fitness…has taken it from just [doing] things to understanding what you're doing. And so that culture change has been very beneficial. [Participant 18]

While participants acknowledged these cultural shifts, many noted that an ongoing emphasis on mission demands continued to affect their daily routines. Specifically, participants described how demanding work schedules often prevented them from prioritizing physical activity to maintain MSK health. They suggested requiring exercise time during the workday, observing that such policies appeared to be implemented in some MOSs, but lacked consistent application across others.

There needs to be a more coordinated effort to provide time during the workday to work out. It is an expectation that we meet a physical fitness standard, but…my workday is 7:30 until 6:30 without time to work out. [Participant 11]

##### 3.2.4.3 Subtheme: prevention vs. reaction

Concerns were voiced by participants that the military's approach to MSK health is primarily reactionary rather than preventive. Participants desired greater investment and education around MSK-I prevention, describing a personal lack of exposure to training on proper movement techniques and form, warm-up and recovery techniques, load carriage preparation (e.g., how to appropriately pack a rucksack), and nutrition to support MSK health. Additionally, participants argued that a stronger emphasis on MSK-I prevention could reduce healthcare costs, expressing a belief that MSK conditions represent a major driver of post-service disability payouts.

Having a formal preventative approach to MSK health would be really helpful…Identifying those units and either placing an embedded provider or providing them access to preventative care…whether that's physical therapy, chiropractic care, providing embedded fitness services…I think that would be hugely beneficial. [Participant 14]

##### 3.2.4.4 Subtheme: intrinsic motivation

Participants emphasized that personal drive plays a significant role in MSK-I prevention and health optimization, observing that some ADSM naturally place a greater emphasis on optimizing their fitness and maintaining good MSK health. For individuals lacking this internal drive, participants suggested that leadership and educational initiatives could help cultivate intrinsic motivation by highlighting the long-term benefits of MSK health, thereby framing fitness as a personal investment, rather than merely a mandatory requirement of military service.

Personally, it's health in general at this point…Beyond the military, I want to live a full life…for service members you need to emphasize most their skeletal resiliency and injury prevention early and often because I think it just makes them more effective individuals. [Participant 56]

#### 3.2.5 Theme 5-leadership: the influence of military leadership on MSK health prioritization and care

Variability in individual leaders' responses to MSK-I was frequently highlighted by participants. While some described supportive leaders, many participants conveyed anecdotes of leaders who were perceived as ignoring participants' MSK-I and medical profiles, actions that participants felt exacerbated their injuries and delayed healing.

My last leader [said] that he doesn't care what I do during the day when doctor's appointments happen, but still need to get my work done…It leads me to working into the middle of the night…bringing work home. [Participant 22]

Reflecting on the hierarchical structure of the military, participants indicated cultural and operational shifts require top-down support. Specifically, they emphasized that command and leadership must actively support positive MSK health—whether by encouraging early care-seeking, respecting medical profiles, promoting MSK-I prevention programs and education, or abiding by physical training guidelines—in order for lasting improvements to occur.

At the end of the day, the mission's going to come first, no matter, regardless of what the injury is…So I think the biggest thing is [going] to depend on what kind of command you're at and it depends what the mission is of that command. [Participant 1]

#### 3.2.6 Theme 6-mental health: the bidirectional relationship between mental and MSK health

Participants described the significant psychological toll of living with a MSK-I or constant MSK-P, reporting that persistent pain exacerbated existing distress or contributed to feelings of depression, anxiety, and stress. Participants frequently expressed that being active and fit was central to their identity, making the loss of physical function or duty capability due to MSK-I/P particularly mentally challenging. Conversely, some participants discussed how existing mental health conditions appeared to influence their experience of MSK-I/P. For instance, one participant noted that as their stress increased, their perceived MSK-P intensified. Encouragingly, a few participants observed a positive shift in military culture toward prioritizing and discussing mental health more openly than in past years.

If you're in chronic pain, it's going to exacerbate any sort of psychological pain. And vice versa. So if your back is constantly hurting, you're probably going to exacerbate any sort of anxiety, depression, concentration issues that you are already experiencing. [Participant 31]

#### 3.2.7 Theme 7-gender: gender-specific experiences and bias in MSK health and care

In addition to epidemiological evidence indicating higher MSK-I rates among female ADSM, interviews revealed gender-specific experiences within MSK health and care, including perceptions of how gender may impact care-seeking and how pregnancy specifically impacts women's MSK health.

A majority of respondents did not perceive that their gender impacted the MSK-I care they received or their decision to seek care. Notably, some men assumed that questions regarding gender applied exclusively to women, suggesting a perception among some respondents that male gender is the default in military settings.

I've been this man my whole life, so I don't know. [Participant 45]

However, a subset of participants spoke to gendered dynamics in care-seeking. One participant suggested that his wife, who is an ADSM as well, avoids seeking care due to past experiences of feeling unheard by providers. This was consistent with the account of a female participant who reported not being believed about her MSK-P, which led her to avoid care in the future. Some participants suggested that women did not want to be seen as a problem or a burden and therefore would avoid seeking care. Another participant remembered that early on in her career, she felt compelled to work extra hard to prove herself and did not want to draw attention to an injury for fear of seeming weak. Other female participants echoed feeling pressure to portray themselves as tough and fit in with their male counterparts. On the other hand, a male participant noted a stereotype of men putting off care, as well, so as not to be perceived as weak, reflecting a broader participant perception that military culture often views MSK-I/P as a sign of weakness, rather than an inevitable fact in a physically active population.

Especially early on in my career, because I was a woman and didn't want to be seen as lesser or weaker…I didn't probably get as much care as I should have until things got really bad. [Participant 26]

Participants with previous pregnancies discussed how pregnancy and postpartum experiences impacted their MSK health. Participants reported experiencing increased ligament laxity, weight gain and postpartum weight retention, and pregnancy-related MSK-I/P such as pelvic girdle and low back pain, core muscle dysfunction, and pubic symphysis dysfunction.

During pregnancy I had pubic separation dysfunction…I was on a wait list [for pelvic floor physical therapy] and I think I'm still on that wait list. So I sought out care on my own and I'm paying out of pocket for it. [Participant 9]

## 4 Discussion

This is the first qualitative study to broadly explore how ADSM navigate MSK health throughout their military careers. The military expects ADSM to maintain exceptional physical readiness and MSK health superior to the general civilian population. Though this standard is realized by some, many ADSM endure suboptimal MSK health, as evidenced by existing quantitative data indicating that MSK-I/P are a leading cause of medical encounters and limited duty days (1). Our results reinforce and expand upon these findings, providing greater context and a richer understanding of the distinct challenges described by ADSM in maintaining MSK health.

### 4.1 Military service and MSK health

Active duty service can be perceived as directly contributing to MSK health, both positively and negatively. On one hand, the emphasis on physical fitness in the military culture fosters a strong drive for maintaining rigorous exercise habits and a team mentality that can encourage an active, healthy lifestyle. Conversely, our interviews revealed that this culture was frequently perceived as promoting a “push through pain” mentality, which participants associated with poor training techniques and increased MSK-I severity. High levels of physical activity without proper recovery, whether due to high operational tempo or self-driven exercise regimens, were also reported as key MSK-I risks, especially when accompanied by improper training techniques. Participants described anecdotes of ADSM attempting intense physical feats without proper preparation, which they felt increased the risk of preventable injuries and delayed healing. According to participant accounts, this cultural dynamic was often reinforced by a perceived organizational prioritization of mission readiness over individual well-being, with some ADSM even deploying with known injuries.

Beyond cultural and systemic pressures, duty-required tasks create repeated physical stressors that take a significant toll on the body, such as heavy load carriage and operating in challenging environments. Previous studies have documented negative effects of military load carriage on functional movement and both upper and lower extremity MSK-I, including pelvic and lower limb stress fracture, neuropathies in the shoulders, thighs, and toes, lower back injuries, and knee and foot pain (20, 21). When these repetitive physical stressors are sustained over the course of a military career, and are compounded by barriers to care-seeking, acute injuries may develop into long-term, debilitating MSK-P. Some participants reported chronic service-related MSK-P so severe that it impacted their daily functioning. Chronic pain, defined as pain that persists for longer than three months, affects 31%–44% of ADSM, is considerably more prevalent among ADSM than civilians, and most commonly stems from MSK conditions. This chronic pain is a leading reason for medical non-readiness and is often comorbid with behavioral health conditions and traumatic brain injury (22). Beyond military service, MSK-P leads to high volumes of VA healthcare utilization, as well, highlighting a need for better pain management and potential resolution during Active Duty service (2, 23, 24).

### 4.2 Care-seeking and the military health system

Although the MHS is designed to be readily available to all ADSM at no cost, seeking care for MSK conditions requires a decision-making process influenced by various barriers, facilitators, and personal factors. While some participants felt the MHS offered better access to care than civilian options, they also highlighted numerous logistical hurdles leading to delayed or avoided care, such as inconvenient clinic locations, an inability to take time off work, and long wait times for appointments, compounded by a complicated and lengthy referral process to see specialists when needed. This aligns with findings among Soldiers in an Army Infantry Brigade Combat Team, where half of those surveyed reported that seeking medical attention is an inconvenience, often leading Soldiers to attempt self-treatment, with leading modalities including the use of over-the-counter pain medication, ice, and heat (9). When care was sought, participants described perceiving inconsistent quality of care for MSK issues and noted frustrations that their first line of care, often a primary care provider, lacked sufficient MSK training. TRICARE was also highlighted as a source of frustration, with participants voicing concerns that billing requirements dictated the terms of care, such as perceiving that they were unable to receive treatment for multiple body parts in a single appointment, even if the issues were related. In participant accounts, this perceived constraint led to a tedious process of multiple appointments, eating up more time and workday hours. This finding echoes the “sluggish” medical care noted in a study of Army Soldiers from over 15 years ago (12), suggesting that despite some improvements, ADSM may still perceive a need for systemic healthcare improvements.

Beyond logistics, fear of career repercussions was prominent in our study, echoing earlier findings from Army and USMC cohorts (7, 8, 13). Participants expressed concern that seeking care or “being put on a profile” would negatively impact their career, mission readiness, or eligibility for certain duties, such as flying, leading to active avoidance of medical care for MSK issues. Although administrative medical retention standards dictate that the physical limitations of a MSK-I itself constitute the formal determinant of duty status, participants in our cohort viewed the act of seeking care as the mechanism that triggers career repercussions, driving care avoidance. Nevertheless, clinical literature emphasizes that early care-seeking should be encouraged, as early treatment may reduce long-term consequences of MSK-I, protecting both the individual's physical health and their career (25). Care-seeking was also described as being discouraged by the perception that being injured signifies failure to contribute to the team or unit. Participants noted not wanting to let their “team” down or feeling like they are a burden when injured. Likewise, among a sample of Army Drill Instructors, 69% agreed that “seeking help would place too much burden on the other Drill Instructors” and 60% agreed that “seeking help would interfere with my ability to train recruits” and these sentiments were both associated with significantly lower odds of reported medical treatment seeking for MSK-I. Furthermore, approximately one-quarter to one-third of the Drill Instructors expressed reluctance to seek help, citing firsthand and secondhand negative experiences seeking medical care in the military alongside skepticism regarding the quality of care available at the local Military Treatment Facility (26). Similarly, some participants in our study believed that seeking care for MSK-I would not be helpful based on previous negative experiences where they felt medical providers did not provide effective treatment, dismissed or disbelieved their pain, or suspected them of exaggerating their symptoms or malingering. These findings suggest that an ADSMs' perceived or prior experience with poor quality healthcare may inhibit continued care utilization. Conversely, a history of experiencing positive provider interactions in the past was reported as making participants more likely to seek care for MSK conditions in the future. Other reported facilitators to care-seeking included easy access to on-site resources like embedded athletic trainers and supportive leadership that actively promoted health and medical care. Data in Army Drill Instructors mirrored our findings, showing that having leadership that promoted healthy behaviors was inversely associated with barriers to care-seeking, including the belief that “seeking help would interfere with my ability to train the recruits,” “seeking help would place too much burden on the other drill sergeants,” and “my leaders would not support me taking time off to see a medical provider” (26).

Even in the absence of overt barriers or facilitators to care, the decision to seek medical attention for a MSK condition is highly personal for all ADSM and requires balancing factors such as pain tolerance, the perceived urgency of the issue, effect on activities of daily living or required duty activities, and the desire to have documentation for future disability benefits. For trainees, deciding to defer care until the end of a training course may reduce the risk of the individual being dropped or recycled through the course, which participants viewed as worthwhile decision if the MSK-I can be tolerated until graduation. A sample of US Army Trainees corroborated this mentality, as “I wanted to graduate on time” was the most endorsed reason for avoiding medical care for a MSK-I during training, with 95% agreeing or strongly agreeing (7). Likewise, if a deployed ADSM is still able to perform his or her duties, deferring care may prevent the team from losing an asset. Conversely, ADSM may be motivated to seek care by the need to establish a “paper trail” for future VA disability benefits. Among a surveyed U.S. Army Infantry Brigade Combat Team, one of the top reasons for seeking care for a MSK-I was “seeking medical documentation” (8). However, participants reported that systemic barriers to care can actively impede ADSM from receiving needed documentation. One participant in our study relayed the irony that ADSM who deploy infrequently and have time to navigate the medical system can accumulate extensive documentation. Meanwhile, ADSM who deploy often and have more physically demanding roles that lead to more MSK issues frequently lack the time to seek adequate medical care leaving them with sparse documentation upon separation and a potentially lower disability rating. The tension surrounding documentation is further complicated by conflicting systemic perceptions. While concerns were raised by some participants that certain individuals may try to over-accumulate documented issues to maximize their disability benefits, others highlighted the opposing perception that MHS providers sometimes try to “pass the buck” by delaying or deferring treatment for chronic issues to the VA. Ultimately, these concerns and ironies illustrate the complex, often contradictory interactions between barriers, facilitators, and personal factors that have dictated MSK medical care-seeking experiences among our participants.

### 4.3 MSK-I prevention vs. MSK health optimization

Several interviews emphasized a need for MSK-I prevention education, with participants suggesting topics such as exercise technique, warm up and recovery strategies, nutrition, and more. Participants also expressed the view that the military's approach to MSK health often seems reactionary rather than preventative. To take that thought a step further, we posit that ADSM MSK health should be about more than just preventing pain and injury; rather, it should be viewed as a continuous spectrum where the goal is MSK health optimization and long-term resilience. Participants suggested that an individual's drive to maintain MSK health plays a vital role in navigating this spectrum, with some ADSM naturally placing greater intrinsic value on fitness. For those lacking this innate drive, participants suggested cultivating motivation through targeted educational initiatives and supportive leadership to foster a culture where ADSM are intrinsically driven by the long-term benefits of MSK health.

While maximizing physical fitness is widely recognized as a critical factor in protecting against MSK-I and optimizing MSK health, there are some caveats. Early-career ADSM, especially those still undergoing rigorous training courses, are often engaged in high levels of physical activity, are more susceptible to injury, and reductions in physical activity volume may be recommended to reduce MSK-I risk (27). Conversely, many of the ADSM who we interviewed were more advanced in their careers and described transitions toward administrative or sedentary work schedules that they felt left insufficient time for physical training, thereby degrading fitness and increasing MSK-I risk. This career-stage distinction is important, as it aligns with prior literature indicating that MSK health promotion efforts must account for distinct subpopulations within the military; one-size-fits-all solutions are rarely successful (28).

While greater fitness and early medical care when needed can prevent the onset and exacerbation of MSK-I, implementing tailored MSK health solutions requires robust infrastructure and resources (29). Participants reported that resources for MSK-I prevention, ranging from fitness centers to athletic trainers, were inconsistent across military installations and service branches. The H2F program provides one such example. H2F is an Army-wide structured initiative to improve readiness across physical, mental, nutritional, sleep, and spiritual domains, but H2F assets are not uniformly available. Recent data highlight the potential value of holistic, system-wide programs, showing that brigades where Army H2F Performance Teams were implemented demonstrated improved metrics with respect to MSK-I profiles, behavioral health profiles, substance abuse, fitness testing, and body composition, plus over $14M in annual cost-avoidance per brigade (30). The investment in H2F illustrates a broader shift within the DoD to view health and fitness through a holistic lens, operationalizing the continuum of care required to foster proactive MSK optimization. However, our qualitative findings alongside existing evidence suggest that the widespread success of such initiatives relies on ensuring ADSM have both the time and administrative support to utilize available resources, something that can be driven by policy changes.

### 4.4 Variable leadership influences MSK health

The views and policies of individual leaders and command were reported to shape MSK health and care-seeking experiences among ADSM. While some participants spoke of supportive leaders who respected medical profiles and encouraged care-seeking, others shared anecdotes of leaders who were perceived to be ignoring injuries, leaving participants feeling forced to continue working and risking further harm. In participant accounts, this disparity was often attributed to a conflict between a leader's responsibility to the mission and their obligation to personnel well-being. For instance, participants described scenarios where upcoming deployments or personnel shortages led leaders to prioritize short-term unit operational capacity over individual injury recovery. Participants noted that this approach varied widely by command and mission, leaving commanders with the challenging task of balancing operational requirements with ADSM health.

Reflecting the hierarchical structure of the military, participants emphasized that cultural and behavioral changes require top-down support. A command that actively promotes injury prevention and respects medical guidance was viewed as essential for fostering a healthier environment. This finding aligns with qualitative evidence showing a negative association between health-promoting leadership scores and barriers to care among Army Drill Sergeants; specifically, as leaders actively prioritized adequate recovery time, emphasized taking care of one's physical and mental health, and encouraged sufficient sleep, Drill Sergeants' perceived barriers to MSK-I treatment-seeking significantly decreased (26). Additionally, reviews of the literature summarizing barriers and facilitators to MSK-I prevention in the military also consistently highlight the necessity of leadership buy-in and support for interventions to be impactful (28, 29). Overall, our findings alongside existing evidence underscore that optimizing MSK health hinges not just on individual actions, but on a health-promoting environment set forth by leadership that effectively balances more immediate operational readiness with long-term health of the ADSM.

### 4.5 MSK and mental health

The connection between the mind and body is a crucial aspect of MSK health. Participants emphasized that living with chronic pain or injury can significantly worsen existing psychological distress, anxiety, and depression. Conversely, participants also mentioned how stress and other mental health conditions can heighten the perception of physical pain, underscoring the need for a holistic approach to care. These sentiments are supported by literature describing the connection between chronic pain and poor mental health, especially among Veterans (31, 32). Research assessing relationships between MSK-I that have not progressed to chronic pain and mental health in Service Members is less robust, though we recently reported that among USMC officers undergoing secondary training, officers with MSK-I/P during training had greater scores on anxiety and depression measures upon graduation from training compared to uninjured officers (33).

For many ADSM, whose identity was described by participants as closely tied to their physical fitness and military role, an injury can be a major source of mental strain. In the athlete literature, a population that is analogous to ADSM in many ways with respect to both physical activity and self-identity, injured athletes often demonstrate higher levels of depression, anxiety, distress, sleep disorders, substance use, post-traumatic stress, disordered eating, emotional disturbances, and feelings of isolation and loss of identity. Furthermore, involuntary retirement from sport due to MSK-I is associated with increased psychological distress, a dynamic that may parallel the experiences of ADSM facing medical separation due to unresolved MSK-I. Numerous psychological and health strategies to combat these negative mental and behavioral health consequences of MSK-I have been evaluated and recommended, such as imagery, goal-setting, coping skills, Mindfulness Based Stress Reduction, Acceptance and Commitment Therapy, and more (34). However, to our knowledge, such strategies are rarely systemically implemented into standard military MSK care. Ultimately, our qualitative accounts alongside literature from athletic and military populations suggest that integrating mental and behavioral health interventions with traditional physical rehabilitation may offer a more comprehensive approach to optimizing military MSK health and recovery.

### 4.6 Gender-specific experiences and bias

The final theme from the interviews revolved around gender and MSK health. While the greater prevalence of MSK-I/P in female ADSM is well-documented (3, 4), our qualitative data provides unique insights into perceived underlying factors contributing to this disparity. Although many participants, both male and female, didn't initially perceive gender as a factor impacting their MSK health, the interviews revealed nuanced, gender-specific experiences. Some female ADSM described feeling the need to work harder and avoid drawing attention to injuries, fearing they would be seen as weak or a burden in a male-dominated environment. This aligns with findings showing that females in Army Initial Entry Training were more likely to endorse not seeking care for a MSK issue to avoid being on a profile and being labeled as injured, broken, or weak (7). Furthermore, women Veterans have reported that that being injured in the military carries a stigma and that they were viewed as weak and less resilient if they admitted to being injured or sought care (14). Despite policy changes in the past decade that have opened all US military roles to women, literature and participant accounts suggest that gender-based stigma can persist and influence aspects of MSK health and care utilization (35).

Female participants described specific MSK challenges related to pregnancy and postpartum recovery, such as pelvic girdle pain and core muscle dysfunction. Physiological and anatomical adaptations to support pregnancy (e.g., ligament laxity, shifted center of gravity, redistribution of blood flow) alongside potential changes in exercise type, frequency, or intensity may increase MSK-I susceptibility during pregnancy and during postpartum recovery. A report on UK Service personnel in Ground Close Combat roles demonstrated that females had a higher number of working days lost due to MSK-I and illness in the 26–52 weeks postpartum compared to pre-pregnancy, with neck and back injuries accounting for the majority of MSK-I (36, 37). Another assessment of British Army personnel reported 71% of postpartum women sustained a MSK-I in the first year after returning to duty compared to 28% of non-postpartum women, with 55% of postpartum injuries occurring in the lumbopelvic, abdominal, hip, and group regions (38). In a qualitative study, females in the Canadian Armed Forces reported that physical training in the postpartum period increased their MSK-I risk and that they faced “unreasonable pressures” to return to pre-pregnancy peak physical readiness despite a shortage of resources and services to support physical recovery (39). Postpartum physical performance degradation is documented in the US military as well. In years past, US ADSM were expected to pass fitness and body composition assessments as soon as six months postpartum. Two Navy studies conducted under those policies reported postpartum Sailors may have reduced core strength, upper body strength, cardiovascular fitness, physical fitness assessment scores, greater BMI, and greater odds of failing physical fitness and body composition assessments within one year postpartum compared to women who did not give birth (40, 41). A qualitative study in postpartum US Airmen provides context for the challenges ADSM face when preparing for their first postpartum physical fitness and body composition assessment, including MSK-P, cesarean wound healing complications, decreased milk supply with intense training, lack of social and emotional support, and the stress of striving to perform well as both an ADSM and mother (42). Although policy changes in the years since these investigations have extended the time frame for postpartum ADSM to return to physical fitness and body composition standards to 12 months, participants in our study highlighted ongoing challenges related to resource access. Specifically, participants noted a lack of accessible specialty care, such as pelvic floor physical therapy, within the MHS. Although each US military service branch has a Pregnancy and Postpartum performance Training (P3T) program, the implementation of these programs was reported to vary by location. A qualitative study of postpartum Army Soldiers corroborated this variability, reporting that the implementation of P3T varied across installations, ranging from helpful to superficial, with some locations not offering the program at all, despite the policy requiring its availability (43, 44). Additionally, Soldiers reported a lack of guidance to return to fitness following delivery (43). We are aware of one military-specific resource, Rehab, Refit, Return to Duty (Rx3), that provides a phased at-home rehabilitation program for postpartum ADSM, though there is a lack of research to date examining the efficacy of the program (45). Our data combined with the existing literature on pregnancy and postpartum ADSM highlights a critical need for additional targeted resources and support for MSK health during this important life stage.

### 4.7 Limitations

This study provides rich qualitative data, but several contextual factors should be considered when judging transferability. First, participants were recruited in the National Capital Region from sites that include major military medical and academic facilities. ADSM in this region may have access to higher-tier facilities and a broader range of specialty services than those stationed at smaller, remote, or resource-constrained installations. Accordingly, findings related to access, referral pathways, specialty care, and facility resources may reflect a comparatively resource-rich or “best-case” MHS context; barriers identified here could differ in type or magnitude elsewhere. However, participants may have drawn upon experiences and anecdotes from other geographic areas they had occupied earlier in their careers as well.

Second, many participants were in medical or medically adjacent MOSs. These participants may have greater health literacy, familiarity with medical terminology, or knowledge of how to navigate the MHS than service members in non-medical occupations, potentially shaping their accounts of care-seeking and system navigation.

Third, we did not collect information on rank. However, the sample had a relatively long military tenure (approximately 16 years on average). Longer-serving participants may have accumulated richer and more varied experiences across duty stations, leaders, and healthcare settings, but their perspectives may differ from those of junior enlisted service members and company-grade officers. Alternatively, this long duration of service may introduce recall bias.

Fourth, although participants represented multiple service branches, the Space Force was not represented.

Rather than limiting the applicability of these findings, these characteristics define the context readers should consider when evaluating transferability to other military settings, ranks, occupations, and career stages. Future qualitative work should intentionally sample across geographic regions, installation types, ranks, years of service, service branches, and medical and non-medical occupations to examine where these themes converge or diverge.

Finally, although our team's complementary expertise in qualitative methodology, kinesiology, and military health enhanced contextual understanding, our civilian status and prior professional background in military and Veteran healthcare research may have subtly influenced data collection, question prioritization, and thematic interpretation. To mitigate this potential limitation, we maintained an ongoing reflexive dialogue throughout analysis, intentionally leveraging our insider familiarity with military health systems alongside our outsider perspective as non-serving civilian researchers to minimize interpretive bias.

### 4.8 Conclusions

In summary, this study provides an in-depth assessment of the complexities of maintaining MSK health in the military, revealing that challenges go far beyond just physical demands of military service to encompass cultural and operational dynamics. Participant accounts highlight potential opportunities to address barriers to care and care-seeking, shift cultural perceptions around MSK-I/P, optimize resource allocation toward prevention, and provide targeted support for ADSM across the MSK health continuum. Based on participant feedback, future efforts could explore strategies the MHS could enact to streamline the referral process for specialty care, reduce wait times, and enhance provider training for common MSK conditions. Leadership initiatives that actively encourage early care-seeking could help prevent injury chronification and contribute to destigmatizing MSK-I/P within unit cultures. Additionally, participants emphasized the potential benefit of expanding unit-level prevention and rehabilitation assets (e.g., embedded athletic trainers) and developing specialized support for female service members during pregnancy and postpartum recovery. Future research can expand on these findings by measuring the prevalence of identified barriers and facilitators across different military installations, branches, and MOSs, and by developing and testing new programs to address these challenges in targeted interventions co-designed with stakeholders to ensure uptake and adherence. By integrating the insights from this study with future research, it will be possible to create more evidence-based tools and resources to help ADSM and medical providers prevent, manage, and treat MSK-I/P more effectively. Ultimately, by addressing these multifaceted challenges through systemic improvements, cultural shifts, and targeted resources, there are opportunities to improve MSK health in ADSM, thereby enhancing readiness and reducing the long-term strains on both MHS and VA.

## Data Availability

The datasets presented in this article are not readily available because of data sharing restrictions on data generated within the United States Department of War. Requests to access the datasets should be directed to emily.ricker.ctr@usuhs.edu.

## Author disclaimer

The opinions and assertions expressed herein are those of the author(s) and do not reflect the official policy or position of the Uniformed Services University or the Department of War. The views expressed in this manuscript are those of the author(s) and do not necessarily reflect the official policy or position of the United States Marine Corps, the Department of the Navy, the Department of War, or the US Government. The contents of this publication are the sole responsibility of the author(s) and do not necessarily reflect the views, opinions or policies of The Henry M. Jackson Foundation for the Advancement of Military Medicine, Inc. Mention of trade names, commercial products, or organizations does not imply endorsement by the US Government.
