# Supplementary material for: Active duty service members' experiences navigating musculoskeletal health throughout their military careers: a qualitative interview study
Source: Front Sports Act Living. 2026 Oct 28;8:1910200. doi: 10.3389/fspor.2026.1910200 (PMC13752063; doi:10.3389/fspor.2026.1910200)
Supplement: Supplementary file 1 [file Supplementaryfile1.docx]

***Supplementary Material***

**Interview Questions**

**Introduction**

Military service can be physically taxing on bones, joints, and muscles, known collectively as the musculoskeletal (or MSK) system. We are interested in learning more about Service Members’ experiences with their MSK health during their military careers. The following questions are related to maintaining your MSK health and physical well-being as a Service Member.

*For your awareness, MSK injuries and pain involve any injury or pain to the bone, muscle, tendon, ligament, and/or cartilage. Further, MSK pain may or may not have been associated with injury.

**Opening Questions**

1. Can you tell me about your experiences with MSK injuries and pain in the past prior to joining the military?
   a. Probe: childhood experiences, pre-military injury
2. Please describe your experience(s) of MSK injury and pain while in the military? [if relevant, skip to facilitators and barriers to care section]
   a. Probe: injury during service, pain during trainings

**MSK Health and Physical Wellbeing: Perceptions of DoD services and programs**

1. In your opinion, in what ways have military duties, equipment, or activities affected your MSK health?
   a. Positively?
      i. Probe: strength training, frequent movement
   b. Negatively?
      i. Probe: sedentary work environment, injury early in career
2. Is having good MSK health a priority for you as a Service Member?
3. Do you use any programs or services offered by [insert branch here] or the military/DoD to help you to maintain or improve your MSK health?
   a. Probe: Rx3, unit PT, access to health and fitness facilities/professionals/programs/resources, H2F (Army side), MCCS or FFIs (Marine Corps side), NOFFS (Navy app), various AT interventions, yoga, nutrition, mindfulness
4. Are there services or programs within [insert branch here] that you don’t currently utilize that could support your MSK health?
   a. What prevents you from using these programs or services?
      i. Probe: lack of time, transportation
5. What would make these programs more appealing to use?
   i. Probe: providing childcare, time off to use services

**Facilitators and Barriers to MSK Health**

1. Does military culture impact MSK health?
   a. Negatively
      i. Probe: lack of time, “suck it up” mentality
   b. Positively
      i. Probe: belonging to the unit, routine
2. Throughout your time in the military, have you learned strategies that helped you to maintain good MSK health and avoid injury?
   a. What did you learn?
   b. When/where/how did you learn it?
   c. What would you have liked to learn more about?

**Script [transition]:** Now we are going to switch gears a bit. Think about a specific MSK injury (or pain) that occurred during your military service. It could be the most serious injury or the one that stands out the most and/or the most frequent or recurrent pain.

**Facilitators and Barriers to MSK Care**

1. Could you describe this injury and/or pain (only give details that you’re comfortable providing)?
2. Did you receive medical care for the MSK-I/P you experienced while in the military? [if no, skip to question 18]
   a. What was the catalyst for your decision to seek care?
      i. Probe: pain, inability to perform at work
3. How soon after the injury or pain began did you seek care?
4. Do you feel you sought care in an appropriate time frame?
   a. Probe: Do you think you should have gone sooner or waited longer?
5. How soon after you sought care did you actually get care?
   a. probe: what was the wait time to see your provider? Were you able to get in to see a provider as soon as you wanted to?
6. What type of provider(s) did you see (initially)?
   a. Probe: medic, PCP, PT, etc.
7. Do you feel that your provider had sufficient knowledge to treat your injury or pain?
8. Did you feel that your provider listened to you and understood your concerns?
9. Did your provider give your injury or pain the attention you felt it deserved? How so?
10. Were you referred to a specialty provider?
    a. Probe: PT/OT/chiropractic care
11. If so, how was that experience?
12. Did you get to see a provider as quickly as you would have liked?
13. Do you feel the specialty care was necessary to treat your pain/injury?
14. Were there things that made it difficult to get care?
    a. Probe: Time, location, family commitments, work commitments
15. Did you have to take time off from training/duties after your injury?
    a. How soon after your injury did you return to duty?
    b. Did you feel you had fully recovered before returning to duty?
    c. What factors influenced your decision or ability to return to duty?
       i. Probe: felt better enough vs. pressure to return
16. Do you think seeking care helped the injury or pain to resolve?
17. Previous research has shown there can be differences in willingness to seek care based on one’s gender. Did your gender play a role in your decision to seek care or not seek care?
    a. Probe: women being seen as weak, men not asking for help [skip to question “closing questions”]
18. [skip if yes to answer 1] If you didn’t seek medical care: why did you choose not to seek medical care?
    a. Did you have any fears of judgment or stigma about having an injury while serving?
    b. Were there geographic barriers, such as transportation issues or lack of specialty care in your area?
    c. What about logistical or supervisory issues?
       i. Probe: being able to take time away from work, training, or family
    d. Did you have to take time off from training/duties after your injury?
       i. How soon after your injury did you return to duty?
       ii. Did you feel you had fully recovered before returning to duty?
       iii. What factors influenced your decision or ability to return to duty?
            1. Probe: felt better enough vs. pressure to return

**Closing Questions**

1. On a 1 to 10 scale, how would you rate the level of importance of MSK health in the [insert military branch here]?
   a. Probe: does the DoD take MSK health seriously, MSK health is vital to mission readiness
2. What would you like to see the [insert military branch here] do to help improve MSK health and prevent injuries among ADSM?
   a. Probe: implement trainings during boot camp on MSK health, emphasize stretching after physical activity

**Background Information**

Age:

Sex:

Military branch:

Time in service:

Occupational specialty:

**Supplemental Table 1. Theme, Subtheme, and Sub-Subtheme Definitions**

| **Theme** | **Subtheme** | **Sub-Subtheme** | **Definition** |
| --- | --- | --- | --- |
| Care-seeking |  |  | A combination of personal, cultural, and systemic factors that influence one's decision to seek or not seek care for a MSK issue |
|  | Barriers |  | Logistical hurdles, mistrust in the system, fear of professional repercussions, and social stigma that prevent one from seeking MSK care |
|  |  | Inconvenience | Logistical and geographic barriers that create difficulty in the process of seeking and receiving MSK care |
|  |  | Career | Negative impact of MSK care-seeking on one's military career often leading ADSM to prioritize professional advancement and a sense of duty over their own physical well-being |
|  |  | Personal | Individual level factors that prevent one from seeking MSK care |
|  |  | Culture | The impact of social stigma, a prioritization of the team over the individual, and a hierarchical structure within the military that keep one from seeking care |
|  |  | Provider | The perceived and/or prior experiences with the quality of the provider-patient interaction that keep one from seeking care or continuing care |
|  | Facilitators |  | A mixture of medical service availability, geographical, social, and systemic factors that promote care-seeking |
|  |  | Access | Logistical and structural conditions that promote MSK care |
|  |  | Culture | Shared beliefs and hierarchical norms that compel individuals to seek care |
|  |  | Personal | Identity and subjective experiences that shape MSK care seeking |
|  |  | Leadership | Leaders encourage ADSM to seek MSK care when needed |
|  | Care decision |  | Factors that influence personal decisions to seek care |
| Military impact |  |  | The effect of military service on MSK health |
|  | Pain |  | Chronic and persistent pain linked to military service |
|  | Service-related issues |  | Direct and indirect physical and operational demands of military life that contribute to MSK health problems |
|  | Culture |  | The impact of the "warrior ethos" on MSK health |
|  | Prioritization |  | The conflict between mission readiness and individual well-being |
|  | Career |  | Fear of the impact that acknowledgement, care-seeking, and/or treatment for an MSK issue would have on one's military career |
| Military Health System |  |  | The combination of access, quality, and patient experience within the healthcare system |
|  | Quality |  | Inconsistent standards and perceptions of care |
|  | Patient opinion of provider |  | The patient's opinion of the provider |
|  | Access |  | The individualized variations on the path to MSK care |
|  | MHS to VA transition |  | Pushing the problem of MSK care to the VA or to be dealt with after service |
| Prevention & optimization |  |  | The systemic and individual barriers and facilitators to MSK health care and maintenance |
|  | Resources |  | The variability in availability, perceived quality, and the utility of MSK tools |
|  | Culture |  | The perceived conflict between military demands and personal health habits |
|  | Prevention v. reaction |  | A deficit in the military's proactive MSK health management |
|  | Intrinsic motivation |  | Personal drive to manage one's MSK health |
| Leadership |  |  | The influence of military leadership on MSK health prioritization and care |
|  | Leader |  | Role of individual leaders in shaping MSK care-seeking behaviors |
|  | Command |  | Conflicts between the mission and individual medical needs |
| Mental health |  |  | The bidirectional relationship between mental and MSK health |
| Gender |  |  | Gender specific experiences and bias in MSK health and care |
